# Targeting multiple pathogenic mechanisms with polyphenols for the treatment of Alzheimer's disease-experimental approach and therapeutic implications

**DOI:** 10.3389/fnagi.2014.00042

**Published:** 2014-03-14

**Authors:** Jun Wang, Weina Bi, Alice Cheng, Daniel Freire, Prashant Vempati, Wei Zhao, Bing Gong, Elsa M. Janle, Tzu-Ying Chen, Mario G. Ferruzzi, James Schmeidler, Lap Ho, Giulio M. Pasinetti

**Affiliations:** ^1^Department of Neurology, Icahn School of Medicine at Mount SinaiNew York, NY, USA; ^2^Geriatric Research, Education and Clinical Center, James J. Peters Veterans Affairs Medical CenterBronx, NY, USA; ^3^Departments of Food Science and Foods and Nutrition, Purdue UniversityWest Lafayette, IN, USA; ^4^Department of Psychiatry, Icahn School of Medicine at Mount SinaiNew York, NY, USA

**Keywords:** Alzheimer's disease (AD), polyphenols, grape seed extract, grape juice, resveratrol, J20 mice

## Abstract

Alzheimer's disease (AD) is the most prevalent neurodegenerative disease of aging and currently has no cure. Its onset and progression are influenced by multiple factors. There is growing consensus that successful treatment will rely on simultaneously targeting multiple pathological features of AD. Polyphenol compounds have many proven health benefits. In this study, we tested the hypothesis that combining three polyphenolic preparations (grape seed extract, resveratrol, and Concord grape juice extract), with different polyphenolic compositions and partially redundant bioactivities, may simultaneously and synergistically mitigate amyloid-β (Aβ) mediated neuropathology and cognitive impairments in a mouse model of AD. We found that administration of the polyphenols in combination did not alter the profile of bioactive polyphenol metabolites in the brain. We also found that combination treatment resulted in better protection against cognitive impairments compared to individual treatments, in J20 AD mice. Electrophysiological examination showed that acute treatment with select brain penetrating polyphenol metabolites, derived from these polyphenols, improved oligomeric Aβ (oAβ)-induced long term potentiation (LTP) deficits in hippocampal slices. Moreover, we found greatly reduced total amyloid content in the brain following combination treatment. Our studies provided experimental evidence that application of polyphenols targeting multiple disease-mechanisms may yield a greater likelihood of therapeutic efficacy.

## Introduction

Alzheimer's disease (AD) is a devastating disorder that strikes 1 in 10 Americans over the age of 65 and almost half of Americans over 85. The odds of developing AD doubles every 5 years after individuals reach the age of 65. There are approximately 35 million US citizens that are 65 years of age or older who are at risk of developing AD, representing 13% of the population. This number will grow dramatically in the coming decades, reaching 17% of the population by the year 2020. Despite tremendous effort to find a cure and effective treatments for the disease, currently available therapies only modestly improve cognitive function and have no effect on disease progression.

Polyphenolic compounds have been increasingly viewed as natural sources for treating numerous illnesses, including neurological disorders, due to their strong antioxidant, anti-inflammatory, anti-microbial, and anti-tumor activities. Mounting evidence suggests that polyphenolic compounds, from a variety of diverse sources, are able to improve cognitive function and reduce brain neuropathology in animal models of AD through multiple mechanisms (Rezai-Zadeh et al., 2005; Ringman et al., 2005; Hartman et al., 2006; Vingtdeux et al., 2008; Wang et al., 2008; Thomas et al., 2009; Wang et al., 2009). Our laboratory and others have shown that grape seed extracts (GSE) rich in proanthocyanidins (PACs) are capable of modulating AD phenotypes through interfering with aggregation of β-amyloid (Aβ) peptides into neurotoxic, soluble high-molecular weight Aβ species (Wang et al., 2008, 2009). We also found that some of the PAC metabolites, such as methyl-epicatechin glucuronide, can effectively promote basal synaptic transmission (BST) and long-term potentiation (LTP) through mechanisms associated with cAMP response element binding protein (CREB) signaling (Wang et al., 2012). Vingtdeux and colleagues demonstrated that dietary supplementation with resveratrol is effective in attenuating the development of Aβ neuropathology in transgenic AD mouse models. Resveratrol may modulate Aβ metabolism through AMP-activated protein kinase (AMPK) signaling mechanism (Vingtdeux et al., 2010). Resveratrol also exerts its anti-inflammatory activity against Aβ-triggered microglial activation via mechanisms involving the TLR4/NF-κ B/STAT signaling cascade (Capiralla et al., 2012). Polyphenol components from red wine and grape juice have also been shown to interfere with Aβ generation and Aβ aggregation (Wang et al., 2006; Ho et al., 2009, 2013). Concord grape juice supplementation in elderly individuals with mild cognitive impairment (MCI) was shown to be beneficial in improving their memory function (Krikorian et al., 2010).

For the past two decades, the majority of AD research has been conducted based on the amyloid hypothesis that deposition of Aβ peptides is the central event for AD pathogenesis (Hardy and Higgins, 1992). Moreover, current drug discovery strategies are mainly targeting various forms of Aβ. The amyloid hypothesis probably holds true for the genetic form of early-onset AD, which accounts for less than 1% of AD cases. However, mechanisms underlying late-onset sporadic AD cannot be explained solely, if at all, by the amyloid hypothesis. Recent genome wide association studies failed to identify polymorphisms in genes associated with APP processing (APP, β- or γ-secretase) and late-onset AD (Gerrish et al., 2012). Moreover, none of the clinical trials targeting various forms of Aβ or APP processing pathways (e.g., Solanezumab, Bapineuzumab, Intravenous Immunoglobulin IVIg, Tramiprosate, Tarenflurbil, Semagacestat) have been successful (Green et al., 2009; Aisen et al., 2011; Doody et al., 2013, 2014; Salloway et al., 2014). It is possible that since neurodegeneration starts 20 years before clinical manifestation, the anti-Aβ intervention is initiated too late to be effective (Selkoe, 2011; Sperling et al., 2011). However, there is increasing realization that AD is not the result of a single cause, but rather multiple mechanisms may synergistically contribute to the onset and progression of the disease (Blennow et al., 2006; Krstic and Knuesel, 2013). For example, inflammation is known to play an important role in AD pathologic mechanisms and is likely a key factor contributing to the onset and progression of AD (Aisen and Davis, 1994; Akiyama et al., 2000; Smith et al., 2000; Krstic et al., 2012; Kumar and Foster, 2013). Krstic et al., demonstrated that chronic inflammation induced by polyriboinosinic-polyribocytidillic acid (Poly I:C) injection led to age-dependent development of an AD-like phenotype in wild type mice (Krstic et al., 2012). More recently, studies demonstrated, in aging rats, that redox regulation can also directly influence N-methyl-D-aspartate receptor (NMDAR) function (Kumar and Foster, 2013), which plays an important role in synaptic plasticity and cognitive function; its function declines during aging and disease condition (Barnes et al., 1997). Individual polyphenolic preparations, such as resveratrol, GSE, or Concord grape juice, have the potential to modulate AD neuropathology and cognitive dysfunction through multiple mechanisms, including modulating oxidation and inflammation, modulating Aβ metabolism, catabolism and oligomerization, or directly influencing brain activities. In this study, we test whether combining these three polyphenolic preparations, with their different polyphenolic compositions and partially redundant bioactivities, provides a safe and effective means to simultaneously and synergistically mitigate Aβ-mediated neuropathology and cognitive impairments in the brain.

## Methods

### Chemicals and materials

Food grade resveratrol was purchased from ChromaDex (Irvine, CA). GSE was purchased from supplement Warehouse (UPC: 603573579173). Only one lot of the resveratrol and one lot of the GSE were used for this particular study. Both resveratrol and GSE have been shown to be very stable when stored at 4°C in dark. Welch Concord purple grape juice was purchased at a local market and concentrated by solid phase extraction (SPE), as previously described, to produce Concord Grape Juice total polyphenol extract (Ho et al., 2009). The polyphenol profile of this juice extract was confirmed by LC-MS analysis and previously reported (Xu et al., 2011). Total polyphenol content was determined to be 62 mg of gallic acid equivalents per mL extract, with principal components including anthocyanins, PAC oligomers, phenolic acids, and quercetin glycosides. The juice extract was stored at −20°C in the dark and was diluted to the desired concentration once every 3 days. (+)-catechin, (−)-epicatechin, quercetin-3-*O*-glucoside, quercetin-3-*O*-glucuronide, and quercetin standards were purchased from Sigma Chemical Co. (St. Louis, MO). Malvidin-3-glucoside chloride and cyanidin-3-glucoside chloride were purchased from ChromaDex (Irvine, CA). All extraction and LC solvents were HPLC certified and were obtained from J.T. Baker (Phillipsburg, NJ).

### Plasma pharmacokinetics (PK) and brain accumulation of polyphenol metabolites following repeated dosing with combination treatment of GSE, concord juice extract, and resveratrol

Plasma PK and brain bioavailability **s**tudies were conducted using male Sprague-Dawley (SD) rats. SD rats were obtained from Harlan Sprague Dawley Inc. (Indianapolis, IN) and placed on a polyphenol free AIN-93M diet (Dyets, Bethlehem, PA), given deionized water ad lib, and allowed to acclimate for 3 days. Following the acclimation period, rats were given a combination of resveratrol, GSE and Concord juice extract (referred to as Comb), by intragastric gavage using plastic feeding tubes (Instech FTP-15-78, Plymoth Meeting, PA) for 10 days. To reach proper dosage, rats were gavaged twice a day, with 8 h separation. The non-treatment group was gavaged with water. Two days prior to PK studies, rats were anesthetized by a dose 3–5% of isoflurane in the anesthesia chamber and maintained with a mask of 1.5–3% isoflurane. A polyethylene catheter was implanted into the jugular vein. Rats were injected with Buprenex (0.01–0.05 mg/kg) before regaining consciousness to alleviate pain and allowed to rest for 24 h after surgery. Catheters were kept patent by flushing with heparinized saline (100 units/mL) every 12 h. Prior to initiation of PK studies, rats fasted for 8 h. PK assessment was conducted on the 10th day of gavage by collecting 400 μ L of blood at baseline (prior to gavage), 0.25, 0.5, 1, 2, 4, 6, 8 h postgavage from the jugular catheter into heparinized tubes. Food was offered at 2 h following initiation of PK studies. Fresh blood was processed to plasma by centrifugation at 5500 rpm for 10 min at 4°C, acidified with saline (1% ascorbic acid wt/v) in 4:1 ratio, purged with N_2_, and stored at −80°C until analysis. The day after PK, rats were given a last dosage of treatment, anesthetized, and perfused with cold physiological saline. The brains were harvested, placed in 0.2% ascorbic acid in saline, and stored at −80°C until analysis.

### Analysis of polyphenol metabolites

Acidified plasma samples were thawed and brought up to 0.5 mL with acidified saline (0.1% formic acid v/v). Polyphenol metabolites from plasma and brains were extracted using SPE as previously described (Vingtdeux et al., 2010; Wang et al., 2012; Ho et al., 2013). Polyphenol metabolites were extracted with methanol and dried under vacuum. Dried residues were reconstituted with 0.1% aqueous formic acid (v/v), loaded on the preconditioned SPE cartridge, eluted with methanol, and dried. The dried phenolic extracts were then reconstituted with 0.1% aqueous formic acid (v/v) and 0.1% formic acid in acetonitrile (v/v) in 4:1 ratio and sonicated, followed by LC-MS/MS analysis.

Analysis of all polyphenol metabolites and quercetin metabolites and PAC derivatives was performed on an Agilent 6400 Triple Quad under multiple reaction monitoring modes (MRM). Both systems were equipped with an ESI source. A Waters XTerra RP-C18 column (2.1 × 100 mm, 3.5 μm particle size) was used on all analysis. A binary mobile phase system, consisting of mobile phase A: 0.1% aqueous formic acid (v/v) and B: 0.1% formic acid in acetonitrile (v/v), was used for analysis of monomeric PACs, resveratrol, and quercetin metabolites. The column was heated to 30°C and the system flow rate was 0.3 mL/min. Gradient conditions were: 10% B at 0 min, 40% B at 5.5 min, 70% B at 7 min, 95% B at 7.5 min and back to 10% B at 8.5 min to 13.5 min. Mass spectra was obtained under negative polarity scanning between 100 m/z-1000 m/z on MS-TOF. MRM mass transitions were 479 → 303 for MeO-EC glucuronides, 465 → 289 for EC-glucuronides, 289 → 137 for EC, 403 → 227 for resveratrol-glucuronide, and 227 → 143 for resveratrol under negative polarity on Triple Quad. MRM mass transitions were 493 → 317 for MeO-quercetin glucuronide, 479 → 303 for quercetin glucuronide, and 301 → 153 for quercetin aglycone under positive polarity. Fragmentor voltage was set at 135V and collision energy was 30 eV for quercetin aglycone and 17 eV for all other mass transitions. ESI source condition was described as followed: gas temp was 350°C, drying gas flow was 11 l/min, nebulizer was 30 psi, sheath gas temp was 350°C, sheath gas flow was 11 l/min, capillary voltage was 3500 V, and nozzle voltage was 1000 V. Quantification of PAC metabolites and resveratrol were estimated using calibration curves from parent catechin, epicatechin, and resveratrol, respectively. Quantification of quercetin metabolites was accomplished using a calibration curve constructed with quercetin-3-O-glucuronide standard. For anthocyanin analysis, the binary mobile phases were A: 2% aqueous formic acid (v/v) and B: 0.1% formic acid in acetonitrile (v/v). The gradient used to elute anthocyanins was: 5% B at 0 min, 10% B at 10 min, 25% B at 30 min, 5% B at 31 min, and continue at 5% B until 35 min. Mass spectra was obtained under positive polarity. ESI source condition setting was the same as described above. MRM transitions were: 493 → 331 for malvidin-3-*O*-glucoside, 479 → 317 for petunidin-3-*O*-glucoside, 465 → 303 for delphinidin-3-*O*-glucoside, 463 → 301 for peonidin-3-*O*-glucoside, and 449 → 287 for cyanidin-3-*O*-glucoside. Quantification of all anthocyanin glucosides, except for cyanidin-3-*O*-glucoside, was estimated using calibration curves of malvidin-3-*O*-glucoside. Quantification of cyanidin-3-*O*-glucoside was achieved by a calibration curve constructed with cyanidin-3-*O*-glucoside standard.

### AD mice and treatment

Female J20 AD transgenic mice, expressing human amyloid precursor protein (APP) containing both the familial AD Swedish (K670N/M671L) and the Indiana (V717F) mutations (APPSwInd) under the human platelet-derived growth factor beta polypeptide (PDGFB) promoter (Mucke et al., 2000), were purchased from the Jackson Laboratory. All mice were housed with food and water available *ad libitum* and maintained on a 12:12-h light/dark cycle with lights on at 07:00° h in a temperature-controlled (20 ± 2°C) room prior to experimental manipulation. All procedures and protocols were approved by the Icahn School of Medicine at Mount Sinai's Institutional Animal Care and Use Committee (IACUC) through the Center for Comparative Medicine and Surgery. Mice were randomly grouped into 5 groups, the non-treated control group (CTRL); the resveratrol-treated group (Resv): 400 mg/kg/day mixed with food; the GSE-treated group (GSE): 200 mg/kg/day mixed with food; the juice extract-treated group (Juice): 183 mg/kg/day total polyphenol; and the group treated with the combination of resveratrol, GSE, and juice (Comb). The doses chosen for each component was based on equivalent doses used in studies that showed efficacy either in human or animal models (Lagouge et al., 2006; Wang et al., 2008, 2010; Krikorian et al., 2010; Vingtdeux et al., 2010). The treatment started at 3 months of age and the animals were treated for 7 months.

### Behavioral assessment of cognitive functions by the morris water maze test

Spatial learning memory was assessed by the Morris water maze (MWM) behavioral test, as previously described (Morris, 1984; Wang et al., 2007). Mice were tested in a circular pool filled with water mixed with non-toxic white paint (Dick Blick Art Materials, IL). In the initial learning phase, mice were trained for 7 consecutive days, which allowed them to learn to escape from the water onto a hidden/submerged (1.5 cm below-water surface) escape platform (14 × 14 cm) in a restricted region of the pool using the spatial cues provided. Spatial learning memory is assessed by recording the latency time for the animal to escape from the water onto the submerged escape platform as a function of the number of learning trials during the learning phase. Twenty-four hours after the last learning session, mice were subjected to a 45 s probe trial wherein the escape platform was removed. Spatial memory retention is reflected by the percentage of time animals spent within the “target” quadrant of the pool that previously contained the hidden escape platform. Water maze activity during training and probe trials was monitored with the San Diego Instrument Poly-Track video tracking system (San Diego, CA).

### Assessment of AD-type amyloid neuropathology

Total Aβ _1−40_ or Aβ _1−42_ in the brain was quantified by sandwich ELISA (BioSource, Camarillo, CA), as previously described (Wang et al., 2005). Plaque burden was analyzed as previously described (Wang et al., 2005). Briefly, tissue was fixed with 4% paraformaldehyde in phosphate buffer and 0.125% glutaraldehyde. Fixed brain hemispheres were sectioned on a Vibratome at 50 μm and stained with thioflavin-S. Micrographs were taken and plaque burden was quantified using Image J software that converts micrograph to binary images for plaque number and plaque area assessment.

### Electrophysiological recordings

Wild type mice were sacrificed by decapitation and the brains were quickly removed. Hippocampal slices (350 μm) were made and placed into oxygenated artificial cerebrospinal fluid (ACSF) at 29°C for a minimum of 90 min to acclimatize. Slices were then transferred to a recording chamber (Fine Science Tools Inc, CA, USA) and perfused continuously with oxygenated-ACSF at 32°C. Slices were treated with 200nM oligomeric Aβ in the presence or absence of 300 nM of 3′-*O*-methyl-epicatechin-*O*-β-Gluc, 300 nM of cyanidin-3-*O*-Glc, or 20 μ M resveratrol for 60 min before transferring to the recording chamber. The use of 20 μ M resveratrol is based on the *in vitro* activation of AMPK study (Vingtdeux et al., 2010). For extracellular recordings: CA1 field excitatory postsynaptic potentials (fEPSPs) were recorded by placing stimulating and recording electrodes in CA1 stratum radiatum as previously described (Gong et al., 2004). BST was not assayed in this particular study because previous studies demonstrated that oligomeric Aβ inhibits LTP in the CA1 region of mouse hippocampal slices without affecting BST (Wang et al., 2002; Olsen and Sheng, 2012). For LTP experiments, a 40–60 min baseline recording period preceded the θ-burst stimulation. Baseline was recorded every min at an intensity that evokes a response ~35% of the maximum evoked response. LTP was induced using θ-burst stimulation (4 pulses at 100 Hz, with the bursts repeated at 5 Hz and each tetanus including three 10-burst trains separated by 15 s) and fEPSPs were monitored for up to 140 min to assess the magnitude of potentiation.

## Statistical analysis

Data was analyzed using SPSS program or Prism software (V4.03, GraphPad Software, Inc, San Diego, CA). Data was presented as mean ± s.e.m. and analyzed using Two-Way ANOVA in a within-subjects design, Two-Way ANOVA with repeated measure, or One-Way ANOVA, followed by Dunnett's multiple comparison, 2-tailed student *t*-test (if sample sizes are equal), or Aspin-Welch procedure (if the sample sizes or standard deviations differed). For electrophysiology study, significant differences were set to ^*^p < 0.05, ^**^p < 0.01, and for others, ^*^p < 0.0125 (*p* < 0.05 adjusting for 4 comparisons), ^**^p < 0.0025 (*p* < 0.01 adjusting for 4 comparisons).

## Results

### Comb treatment does not change plasma PK response or brain profile of polyphenol metabolites

To test whether polyphenols delivered in combination would change the brain bioavailability, we treated the SD rats with Comb and assessed the plasma PK and brain polyphenol metabolites' profiles and compared these profiles with those from previous studies on individual polyphenol preparation (Vingtdeux et al., 2010; Wang et al., 2012; Ho et al., 2013). Consistent with previous findings (Wang et al., 2008; Vingtdeux et al., 2010), all metabolites were found in the plasma and brain following 10-day Comb treatment: catechin and epicatechin from GSE led to the accumulation of PAC glucuronides (Gluc) including catechin-*O*-β-Gluc, 3′-*O*-methyl-catechin-*O*-β-Gluc, epicatechin-*O*-β-Gluc, and 3′-*O*-methyl-epicatechin-*O*-β-Gluc; quercetin from juice resulted in the accumulation of quercetin-Gluc, *O*- methyl quercetin-Gluc, malvidin-glucoside (Glc), petunidin-Glc, delphinidin-Glc, and peonidin-Glc; cyanidin-Glc from juice led to the plasma and brain accumulation of malvidin-3-*O*-glucoside (Glc), petunidin-3-*O*-Glc, delphinidin-3-*O*-Glc, peonidin-3-*O*-Glc, and cyanidin-3-*O*-Glc; resveratrol led to the accumulation of resveratrol and resveratrol Gluc in the brain. See Table 1 for detailed plasma PK response and brain accumulation of polyphenol metabolites in rats treated with Comb.

**Table 1:** **Plasma pharmacokinetics and brain accumulation of polyphenol metabolites**.

**Polyphenol composition in Comb**	**Concentration**		**Metabolite**				**Normalized by Dose**		
	**(mg/g)**	**Dose (mg)**		**Cmax (umol/L)**	AUC (umol/L*h)	**Brain (pmol/g)**	**Cmax (uM/mg)**	AUC (uM*h/mg)	**Brain (nM/mg)**
Catechin	5.6	2.78	Catechin-5-0-Glucuronide	2.46 ± 0.26	7.89 ± 0.19	485.79 ± 85.07	0.886	2.842	174.966
	5.6	2.78	3′0Me-Catechin-5-0-Glucuronide	2.82 ± 0.07	12.57 ± 0.56	664.29 ± 133.65	1.016	4.527	239.256
Epicatechin	6.49	3.21	Epicatechin-5-0-Glucuronide	2.79 ± 0.19	9.12 ± 0.30	637.22 ± 93.85	0.867	2.834	198.033
	6.49	3.21	3′0Me-Epicatechin-5-0-Glucuronide	4.20 ± 0.13	18.46 ± 0.47	853.83 ± 142.77	1.305	5.737	265.351
Quercetin	0.09	0.044622	Quercetin-3-0-Glucuronide	0.11 ± 0.04	0.39 ± 0.073	2.41 ± 0.47	2.658	8.636	54.009
	0.09	0.044622	OMe-Quercetin-O-Glucuronide	0.079 ± 0.008	0.41 ± 0.044	0.69 ± 0.05	1.766	9.191	15.463
Malvidin glucoside	0.014	0.0069412	Malvidin glucoside	0.004 ± 0.0004	0.016 ± 0.044	0.17 ± 0.02	0.602	2.294	24.491
Petunidin glucoside	0.015	0.007437	Petunidin glucoside	0.003 ± 0.0005	0.020 ± 0.004	0.10 ± 0.00	0.418	2.668	13.446
Delphinidin glucoside	0.03	0.014874	Delphinidin glucoside	0.004 ± 0.0003	0.017 ± 0.005	0.07 ± 0.00	0.282	1.129	4.706
Peonidin glucoside	0.01	0.004958	Peonidin glucoside	0.004 ± 0.0002	0.017 ± 0.0005	0.12 ± 0.01	0.785	3.507	24.203
Cyanidin glucoside	0.023	0.0114034	Cyanidin glucoside	0.01 ± 0.001	0.010 ± 0.0015	0.07 ± 0.00	0.843	0.901	6.139
Resveratol	114	56.5212	Resveratrol-3-0-Glucuronide	78.53 ± 3.24	456.44 ± 75.98	746.57 ± 121.73	1.389	8.076	13.209

*Brain polyphenol metabolites analysis following 10 days repeated dosing of combination of GSE, concord grape juice extract and resveratrol*.

### Comb treatment improves special memory function

**Figure 1:**
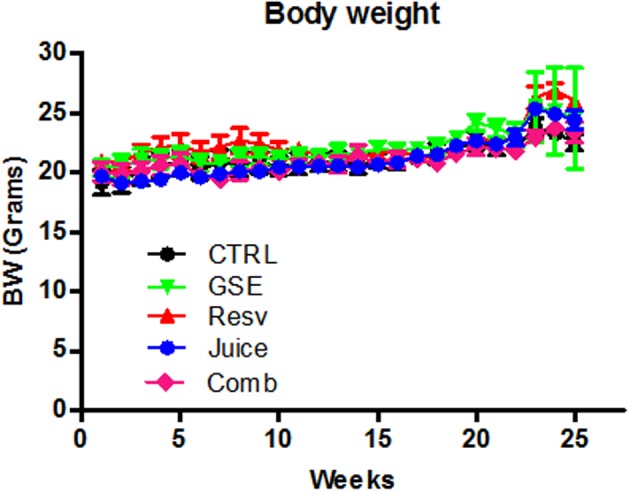
**Chronic Comb treatment has no adverse effect body weight measurements over the 5 month dietary treatment**. Data represents mean ± s.e.m., *n* = 8 – 9 per group.

We treated J20 mice with Resv, GSE, Juice, or Comb, starting at 3 months of age. Treatments continued for 7 months until mice were approximately 10 months of age. Consistent with our previous studies (Wang et al., 2008; Vingtdeux et al., 2010), we found that polyphenolic treatment from all groups were well-tolerated; we observed no adverse effects in response to long-term treatments as reflected by normal drinking, eating, grooming behavior, and normal body weight (Figure 1).

**Figure 2:**
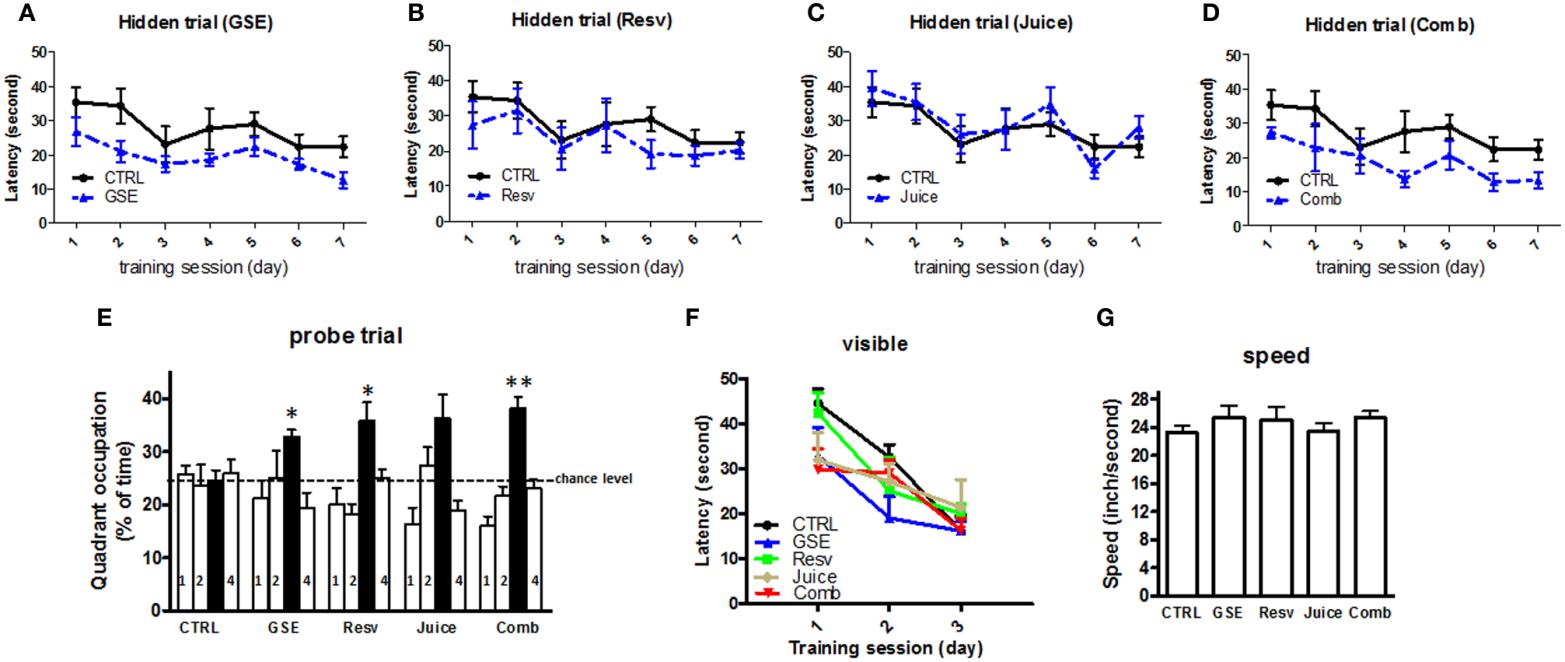
**Chronic Comb treatment attenuates cognitive deterioration in J20 mice. (A–D)** The influence of chronic GSE, Resv, Juice, or Comb treatment on Aβ-related spatial memory in J20 mice vs. untreated control J20 mice in the Morris water maze test. Hidden platform acquisition: latency score represents the time taken to escape to the platform from the water **(E)** Spatial memory retention in the probe trial: percent of time in quadrant is calculated as the ratio of time spent in the target quadrant area relative to the time spent in the rest of the pool (quadrants: : target; 1-left; 2: opposite; 4: right) **(F)** Cued platform visible trial **(G)** Average swimming speed. Data represents mean ± s.e.m. *n* = 7 – 9 per group.

The MWM test was used to evaluate cognitive function following treatment. In the hidden platform learning trial, we found GSE treatment significantly improved the cognitive behavioral performance of J20 mice, in comparison to age- gender-matched non-treated, CTRL, J20 mice [*F*_(1, 13)_ = 3.726, *p* = 0.038 for GSE treatment effect, Two-Way ANOVA RM, Figure 2A]. Neither Resv nor Juice treatment led to significant improvements in MWM training trial (Figures 2B,C). The Comb mice also performed significantly better than the CTRL mice [*F*_(1, 14)_ = 6.627, *p* = 0.0231 for treatment effect, Two-Way ANOVA RM, Figure 2D].

Interestingly, compared to the CTRL mice, all groups of treated J20 mice, except the Juice group, performed significantly better in the probe trial phase of the MWM test 24 h after the last training session (One-Way ANOVA, *p* = 0.021; GSE vs. CTRL, ^*^p < 0.012; Resv vs. CTRL, ^*^p < 0.012; Comb vs. CTRL, ^**^p < 0.001, Figure 2E and Table 2 for statistical analysis). Juice group performed better than the CTRL group, but did not reach statistical significance (*p* = 0.032, Figure 2E). Our data suggest that GSE, Resv, Comb, and possibly Juice treatment lead to significant improvement in spatial memory retention. In parallel control studies (Figures 2F,G), we confirmed that all five groups of J20 mice performed equally well, excluding the possibility that the polyphenol treatments might have affected non-spatial parameters, such as sensorimotor performance and motivation, which might have interfered with behavioral performance during the MWM test.

**Table 2:** **Significance of comparisons of the control treatment with GSE, Resv, Juice, or Comb treatments**.

**Compared to CTRL**	**Probe trial (memory retention)**	**Aβ_1–42_**	**Aβ_1–40_**	**Amyloid plaque**
Grape seed extract	0.012^*^	0.023	0.020	0.023
Resveratrol	0.012^*^	0.968	0.284	0.892
Juice polyphenol extract	0.037	0.741	0.432	0.797
Combination treatment	0.001^*^	0.001^**^	0.007^*^	0.015

*Statistical analysis of data from the probe trial, brain A^β^, and plaque burden to compare the treatment groups vs. the control group*,

* p < 0.0125 (p < 0.05 adjusting for 4 comparisons);

** *p < 0.0025 (p < 0.01 adjusting for 4 comparisons)*.

### Differential effect of polyphenol metabolites on oAβ induced acute synaptic dysfunction

**Figure 3:**
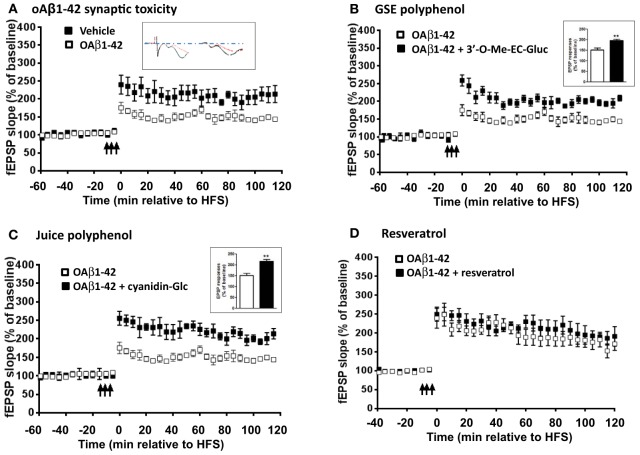
**Select brain-penetrating polyphenolic metabolites from GSE and Concord grape juice are bioactive in preventing acute oAβ-induced LTP impairment**. *Ex vivo* hippocampal slices from wild type (WT) mice were acclimated in oxygenated artificial cerebrospinal fluid and challenged with **(A)** 200 nM of oAβ **(B)** 200 nM oAβ co-treated with 3′-*O*-Me-EC-Gluc (300 nM), **(C)** 200 nM oAβ co-treated with cyanidin-Glur (300 nM) and **(D)** 200 nM oAβ co-treated with resveratrol (20 μ M) for 1 h before recording. The fEPSPs were recorded from the CA1 region. The fEPSP slopes (% of baseline) were plotted as a function of time. The arrows indicate the beginning of tetanus used to induce LTP. Inset in **(A)**, representative EPSP traces for vehicle (left) and oAβ (right) treated slices: Red trace represents pre-LTP and black trace represents 60 min following tetanus stimuli. Insets in **(B)** and **(C)**, average magnitude of LTP during the last 5 min of recording. Data represents mean ± s.e.m., ^**^P < 0.01.

LTP is one of the major cellular mechanisms that underlies synaptic plasticity and is critical for learning and memory (Bliss and Collingridge, 1993; Cooke and Bliss, 2006). Based on our observation that Resv and Juice treatment improved memory retention in the J20 mice, we explored the possibility that accumulation of polyphenol metabolites in the brain, following chronic polyphenol treatment, might directly contribute to the cognitive benefits through the promotion of LTP. We tested three metabolites that have been identified in the brain following different dietary polyphenol treatment in modulating oAβ induced LTP deficits: 3′-*O*-Me-EC-Gluc from GSE, cyanidin-Glc from Concord grape juice, and resveratrol. It is well established that oAβ are potent synaptotoxins and can impair synaptic plasticity. Hippocampal slices isolated from young mice exhibited normal LTP response following tetanus stimulation (Figure 3A), while 1-h bath perfusion of 200 nM oAβ completely inhibited LTP (Figure 3A). We found that 3′-*O*-Me-EC-Gluc and cyanidin-Glc, applied at nanomolar concentrations, significantly protects against acute oAβ-mediated LTP deficits (Figures 3B,C). Co-treatment with 3′-O-Me-EC-Gluc resulted in significantly increased LTP, expressed as a percentage of baseline fEPSP slope compared with the vehicle treatment (196.7 ± 6.5% vs. 151.8 ± 9.5 %, *p* < 0.01, Two-Way ANOVA). Co-treatment with cyanidin-glc resulted in a significantly increased LTP, expressed as a percentage of baseline fEPSP slope compared with the vehicle treatment (216.7 ± 8.5 % vs. 151.8 ± 9.5 %, *p* < 0.01, Two-Way ANOVA). In contrast, the application of resveratrol did not protect against oAβ-induced synaptic deficits (Figure 3D). This observation revealed that select brain-targeted metabolites from GSE and Concord grape juice extract are capable of directly protecting against AD-type oAβ-mediated synaptic toxicity in the hippocampal formation and may contribute to the improved cognitive function observed in the J20 mice.

### Comb treatment reduces brain amyloid neuropathology

**Figure 4:**
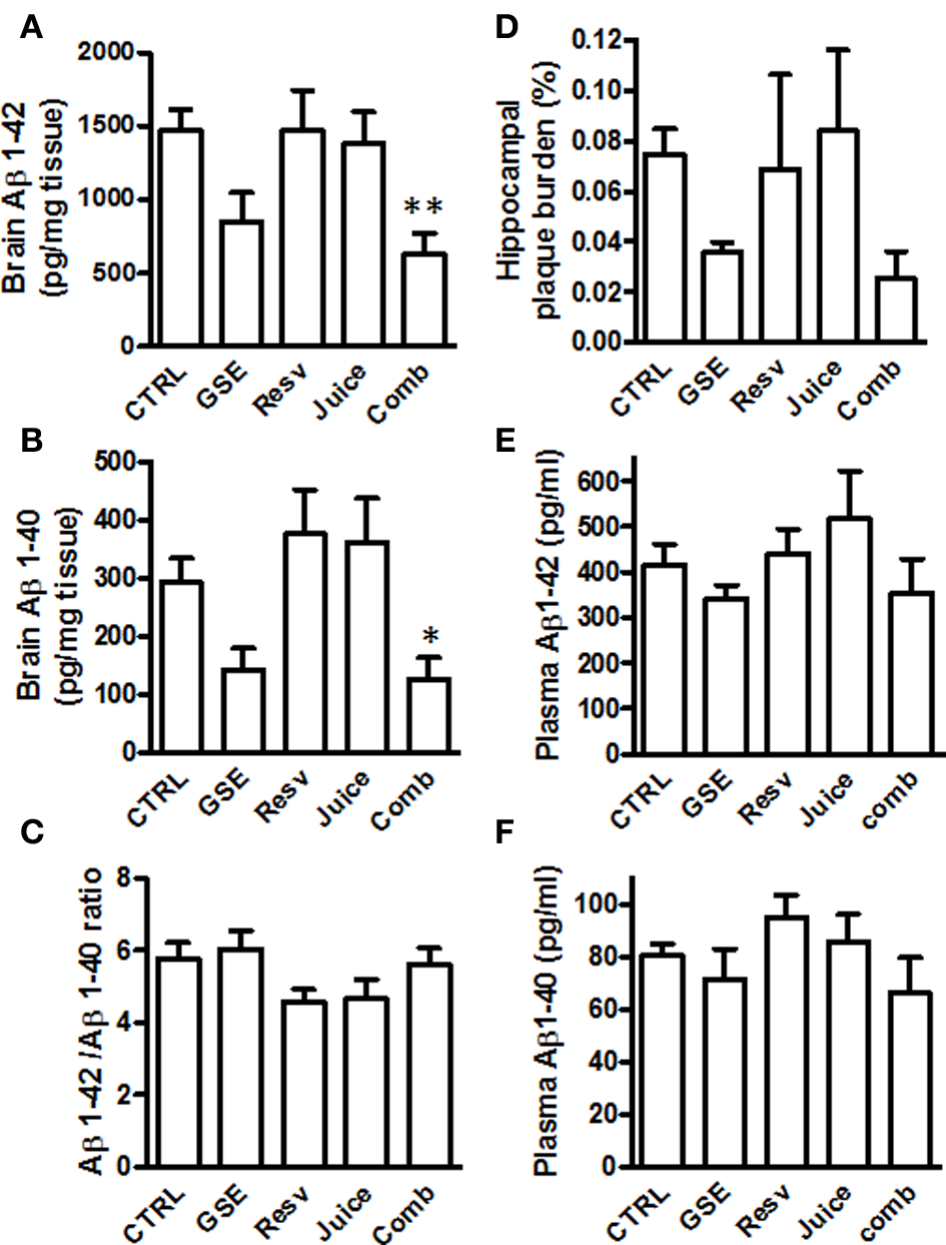
**Chronic Comb treatment reduces brain neuropathology in J20 mice (A,B) Quantification of total Aβ_1–42_ and Aβ_1–40_ in the brains of GSE-, Resv-, Juice-, Comb-treated, or CTRL mice by ELISA measurements **(C)** The ratio of Aβ_1–42_ to Aβ_1–40_ in the brains of GSE-, Resv-, Juice-, Comb-treated, or CTRL mice**. **(D)** Assessment of plaque burden (percentage of area covered by plaques) in the hippocampus of the J20 mice following chronic treatment. **(E,F)** Assessment of plasma levels of amyloid peptides by ELISA. Data represents mean ± s.e.m., *n* = 8 – 9 per group.

Following MWM behavior assessment, we sacrificed the animals and evaluated the impact of the different treatments on Aβ in the brain. We found that both GSE and Comb treatment led to a reduced Aβ _1−42_ content in the brains, however, only the Comb group reached statistical significance [One-Way ANOVA, *p* = 0.0098; Comb vs. CTRL, ^**^p < 0.001, *F*_(1, 14)_ = 4.354, Figure 4A and see Table 2 for statistical analysis]. Neither Juice nor Resv treatment had any effect on brain Aβ _1−42_ levels (Figure 4A). Similar results were obtained for the levels of Aβ _1−40_ [One-Way ANOVA, *p* = 0.0067; Comb vs. CTRL, ^*^p < 0.007, *F*_(1, 14)_ = 3.152; Figure 4B and see Table 2 for statistical analysis]. Aβ _1−42_ peptides are more prone to aggregate and it is believed the increased ratio of Aβ _1−42_/Aβ _1−40_ may contribute to the pathogenesis of the disease. We found that polyphenol treatment resulted in a reduced Aβ _1−42_/Aβ _1−40_ ratio in the brain (One-Way ANOVA, *p* = 0.0112, Figure 4C), and both Juice and Resv groups showed a strong trend of reduced ratio of Aβ _1−42_/Aβ _1−40_ in the brain compared to the CTRL group (*p* = 0.06 for both groups). Plaque burden analysis revealed that GSE treatment and Comb treatment reduced the load of plaques in the hippocampal formation compared to the CTRL group, while Resv and Juice had no effect (Figure 4D and see Table 2 for statistical analysis). Plasma amyloid peptide measurements showed that none of the treatments had any effect on the levels of amyloid peptides in the plasma (Figures 4E,F).

## Discussion

AD is a multifaceted disease and the etiology is largely unknown. It is clinically characterized by progressive and degenerative memory loss and cognitive function, and pathologically characterized by extracellular accumulation of neuritic plaques made of Aβ protein and neurofibrillary tangles (NFTs) composed of tau proteins in the brain. AD is one of the most persistent and devastating neurological disorders, and there is no effective treatment. Due to the complexity of the disease and the limited knowledge of how the disease starts and progresses, the search for a cure has not been successful, despite tremendous efforts rendered by both academic institutions and the pharmaceutical industry, with over 1000 clinical trials. There is a growing consensus that the failure to develop an effective intervention for AD may be due, in part, to the fact that almost all past preclinical and clinical trials have been designed to target individual pathological features, which in the past two decades, mainly focus on various forms of Aβ and APP processing. It is hypothesized that novel intervention regimen(s) designed to simultaneously interfere with multiple pathological features may yield a greater likelihood of therapeutic efficacy.

In recent years, there is increasing interest to explore the potential value of plant derived polyphenols in treating neurological disorders, including AD. There is accumulating evidence that some of the pathophysiological effects associated with metabolic syndrome are beneficially modulated by dietary supplementation of polyphenols (Marambaud et al., 2005; Rezai-Zadeh et al., 2005; Ehrnhoefer et al., 2008; Ono et al., 2008; Rocha-Gonzalez et al., 2008; Wang et al., 2008; Karuppagounder et al., 2009; Thomas et al., 2009; Krikorian et al., 2010; Ho et al., 2011; Liu et al., 2011; Capiralla et al., 2012). Majority of these studies were focused on grape derived polyphenols, including resveratrol. One of the major limitations of polyphenolic compounds for treating neurological disorders is their complicated absorption, interaction, metabolism, and eventually their bioavailability in targeted organs, mainly the central nervous system. We carried out a brain bioavailability study to explore whether oral administration of the combination of GSE, Resv, and Juice would alter the metabolism and brain penetration of the compounds and their metabolites. We found that all the metabolites identified in the brains, through individual compound administration, were also found in the brains of the rats treated with the Comb, suggesting that these three compounds did not interfere with each other to alter their absorption, metabolism, and brain penetration.

We compared the spatial memory function of J20 mice treated with Comb or individual compounds to explore whether Comb treatment can render additive or synergistic effects to improve cognitive function. Consistent with our previous GSE study (Wang et al., 2008), both GSE and Comb groups performed well in learning trials, while the Resv and the Juice group did not show appreciable improvements. All groups performed better than the non-treated CTRL mice in the probe trial, with the Comb treatment group showing slightly better performance than the groups treated with individual polyphenols. Neuropathology analysis also showed that the Comb group had much lower amyloid content and plaque burden compared to the CTRL mice. However, the Resv and Juice had no effect on amyloid neuropathology in the hippocampal formation. Our data seems to be consistent with the observation reported by Vingtdeux et al. (2010) that chronic resveratrol treatment does not reduce plaque deposition in the hippocampus. However, their study demonstrated that resveratrol treatment reduced both sodium dodecyl sulfate (SDS) and formic acid fraction of Aβ in the brain. It is possible that this difference is due to the different strains of mice used (APP/PS1 vs. J20) or a different treatment regimen. To investigate the improvements of memory retention in the GSE, Resv, and Juice treatment, we used electrophysiology to measure LTP, one of the important mechanisms for long-term memory. We found that select polyphenol metabolites from GSE and Juice significantly improved acute oAβ-mediated LTP deficits in hippocampal slices. Surprisingly, resveratrol, previously shown to be able to increase phosphorylation of CREB (phosphor-Ser-133), an important molecule involved in memory consolidation, did not show any effect in restoring acute oAβ-mediated synaptic toxicity.

In conclusion, our studies demonstrated that when delivered in combination, the potentially bioactive metabolites from GSE, resveratrol, and Concord juice extract can pass the blood brain barrier and accumulate in the brain. We found that Comb treatment provided better protection against amyloid neuropathology and better preservation of cognitive function. We deduce that the anti-amyloidogenic activities of the Comb treatment may be mainly derived from GSE, while both GSE and Juice may contribute to the improved cognitive function by improving synaptic plasticity. There are many other amyloid-independent mechanisms targeted by the individual polyphenol preparation that may collectively contribute to improved cognitive function and overall brain health. Both juice and resveratrol have strong antioxidant and anti-inflammatory activities and can potentially inhibit amyloid-mediated microglia activation (Capiralla et al., 2012) or modulate inflammation-mediated blood brain barrier dysfunction. However, the J20 mice used in the present study and other existing mouse models of amyloid pathology are inadequate to address these questions, as they do not have similar, or as extensive, inflammatory processes as humans. Future studies using appropriate models, such as inflammation induction by injection of Poly I:C (Krstic et al., 2012) to both wild type and APP mice, might be able to help us determine the anti-inflammation activities of bioactive polyphenols and their role in AD disease therapy.

Besides age and genetics, many factors contribute to the onset and progression of AD. While Aβ proteins and NFTs are certainly major culprits, oxidative stress and inflammation play important roles in the pathogenesis of AD (Aisen and Davis, 1994; Akiyama et al., 2000; Smith et al., 2000; Krstic et al., 2012; Kumar and Foster, 2013; Lee et al., 2013). Given the increasing consensus that multiple mechanisms during aging may synergistically contribute to AD pathogenesis and cognitive decline, it is important to continue investigating the role of combination therapies in modulating synaptic plasticity, including BST and LTP, mitochondrial function, oxidative stress, and inflammation in models of AD and aging, including experimental models of amyloid, tau, chronic inflammation, and oxidative stress. Our future studies will focus on clarifying these mechanisms and continuing to identify specific bioactive metabolite(s) and their mechanisms of action for future application in preventing and treating AD and other forms of dementia.

### Conflict of interest statement

The authors declare that the research was conducted in the absence of any commercial or financial relationships that could be construed as a potential conflict of interest.
